# Impact of *NUDT15* polymorphisms on thiopurines-induced myelotoxicity and thiopurines tolerance dose

**DOI:** 10.18632/oncotarget.14594

**Published:** 2017-01-11

**Authors:** Dandan Yin, Xuyang Xia, Junlong Zhang, Shouyue Zhang, Fei Liao, Ge Zhang, Yan Zhang, Qianqian Hou, Xue Yang, Hong Wang, Zhigui Ma, Heyao Wang, Yiping Zhu, Wei Zhang, Yuelan Wang, Bo Liu, Lanlan Wang, Heng Xu, Yang Shu

**Affiliations:** ^1^ Department of Laboratory Medicine, National Key Laboratory of Biotherapy/Collaborative Innovation Center of Biotherapy and Precision Medicine Key Laboratory of Sichuan Province, West China Hospital, Sichuan University, Chengdu, Sichuan, China; ^2^ Department of Laboratory Medicine/Research Center of Clinical Laboratory Medicine, West China Hospital, Sichuan University, Chengdu, Sichuan, China; ^3^ Department of Pediatric Hematology/Oncology, West China Second Hospital Sichuan University, Chengdu, Sichuan, China; ^4^ Department of Thoracic Oncology, Cancer Center, National Key Laboratory of Biotherapy, West China Hospital, Sichuan University, Chengdu, Sichuan, China; ^5^ Integrated Biomedical Sciences Program, University of Tennessee Health Science Center, Memphis, TN, USA; ^6^ Department of Precision Medicine, China-Japan Friendship Hospital, Beijing, China; ^7^ Department of Clinical Pharmacology, Hunan Key Laboratory of Pharmacogenetics, Xiangya Hospital, Central South University, Changsha, China

**Keywords:** meta-analysis, NUDT15, thiopurines-induced myelotoxicity, intolerance dose

## Abstract

Thiopurines are widely used as anticancer and immunosuppressive agents. However, life-threatening myelotoxicity has been noticed and largely explained by genetic variations, including *NUDT15* polymorphisms (e.g., rs116855232). In this study, we conduct a meta-analysis to investigate the impact of rs116855232 on thiopurines-induced myelotoxicity susceptibility (1752 patients from 7 independent cohorts), as well as on thiopurines intolerance dose (2745 patients from 13 cohorts). Variant allele of rs116855232 contributes 7.86-fold (*P* < 0.00001, 95% CI: 6.13–10.08) higher risk to develop leucopenia with high specificity (91.74%) and sensitivity (43.19%), and lower thiopurines intolerance dose (*P* < 0.00001). Through bioinformatics prediction, amino acid changes induced by genetic variants are considered to reduce the stability, and break an α helix of NUDT15, which is part of the thiopurine binding pocket. Additionally, we conduct an expression quantitative trait loci (eQTL) analysis for *NUDT15*, and find a promoter-located eQTL signal (rs554405994), which may act as a potential marker to predict thiopurines-induced myelotoxicity. In conclusion, genetic polymorphisms in *NUDT15* are strongly associated with adverse drug reaction (ADR) of thiopurines, although more evidences are needed to determine values of all functional *NUDT15* polymorphisms for clinical regimen, rs116855232 should be considered as a highly credible pharmacogenetic indicator for thiopurines using espcially is Asians.

## INTRODUCTION

As immunosuppressive and anticancer pro-drug, thiopurines (e.g., azathioprine [AZA], mercaptopurine [6-MP]), alone or in combination with other agents, remain a gold standard medical therapy for the maintenance of disease remission in patients with acute lymphoblastic leukemia (ALL), inflammatory bowel diseases (IBD), and so on [1, 2]. AZA and 6-MP can convert to active metabolites 6-thioguanine nucleotides (6-TGNs) via multiple sequential anabolic reactions [3, 4], which (e.g., deoxythioguanosine triphosphate) can incorporate into double-stranded DNA to trigger futile mismatch repair and lead multiple types of cells (e.g., T lymphocytes) to apoptosis and subsequent resolution of inflammation [5–8]. Meanwhile, 6-MP and its metabolites can be methylated by thiopurine methyltransferase (TPMT) and interrupt intervening DNA synthesis [9, 10]. Therefore, TPMT activity has been noticed to be negatively related to 6-TGN content in plasma [11].

Large interindividual variations in dose responses and ADR susceptibility has been noticed due to the narrow therapeutic index of thiopurine drugs, with common dose-dependent toxicities, including myelotoxicity and hepatotoxicity [8, 12]. One well reported and accepted explanation is that patients with TPMT deficiency induced by genetic variants can increase sensitivity to myelotoxicity effects of thiopurine drugs [13, 14]. Actually, several single nucleotide polymorphisms (SNPs) in *TPMT* gene (e.g., rs1142345) have been labeled as pharmacogenetic markers, and strongly recommended to be genotyped for clinic usage of thiopurines [15–18]. However, racial diversity of *TPMT* SNPs in terms of variant allele frequencies limits their prediction values. For example, rs1142345, which is the most common *TPMT* SNP (also indicated as TPMT*3C), has allele frequency of 4% in Caucasians, but only 1.3% in East Asians. Paradoxically, thiopurines-induced leukopenia is more common in Asians, and quite a few patients with wild-type *TPMT* are intolerant to full dose of thiopurine drugs [19, 20], suggesting the existence of other underlying race specific genetic polymorphisms in thiopurine response. Recently, two independent studies have identified a variant in *NUDT15* gene (i.e., rs116855232, inducing p.Arg139Cys) to be associated with intolerance to thiopurines or thiopurines-induced ADR in patients with ALL and IBD, respectively [2, 12]. Such association has been replicated by multiple independent studies [14, 21–28], and expanded to several other *NUDT15* SNPs, including rs147390019 (inducing p.Arg139His) [24]. Large genetic population studies (e.g., ExAC project) demonstrate that variant allele of rs116855232 of *NUDT15* is most common in East Asians (10.4%) and Hispanics (7.1%), rare in Europeans (0.46%), but barely detected in Africans, while rs147390019 is mostly in Hispanic (1.75%) [29], contributing to ancestry-related differences in thiopurine drugs tolerance [12, 19, 30].

NUDT15 is deemed to dephosphorylate the thiopurine active metabolites TGTP and TdGTP, preventing their incorporation into DNA and negatively affecting the cytotoxic effects of thiopurines [2, 3, 14, 21–24, 28, 31–33]. Crystal structure of NUDT15 has been characterized, making it possible to estimate the impact of Arg139Cys and Arg139His on NUDT15 activity, and subsequent cell sensitivity to thiopurine treatment. Indeed, *in vitro* pharmacological analyses and cellular drug response examinations have been done and determined the NUDT15 deficiency induced by not only genetic variants, but also the expression level of *NUDT15* [24], highlighting the importance of *NUDT15* SNPs genotyping for clinic use of thiopurine drugs.

In this study, we aim to conduct a systematic review and meta-analysis to investigate the association of *NUDT15* SNPs with clinic thiopurine response on the basis of existing researches, and examine the impact of these common variants on NUDT15 structure through bioinformatics analyses. Finally, eQTL analyses are proceeded to search more pharmacogenetic markers for thiopurine induced ADR in *NUDT15* gene, in order to increase the prediction sensitivity.

## RESULTS

### Meta-analyses

**Figure 1:**
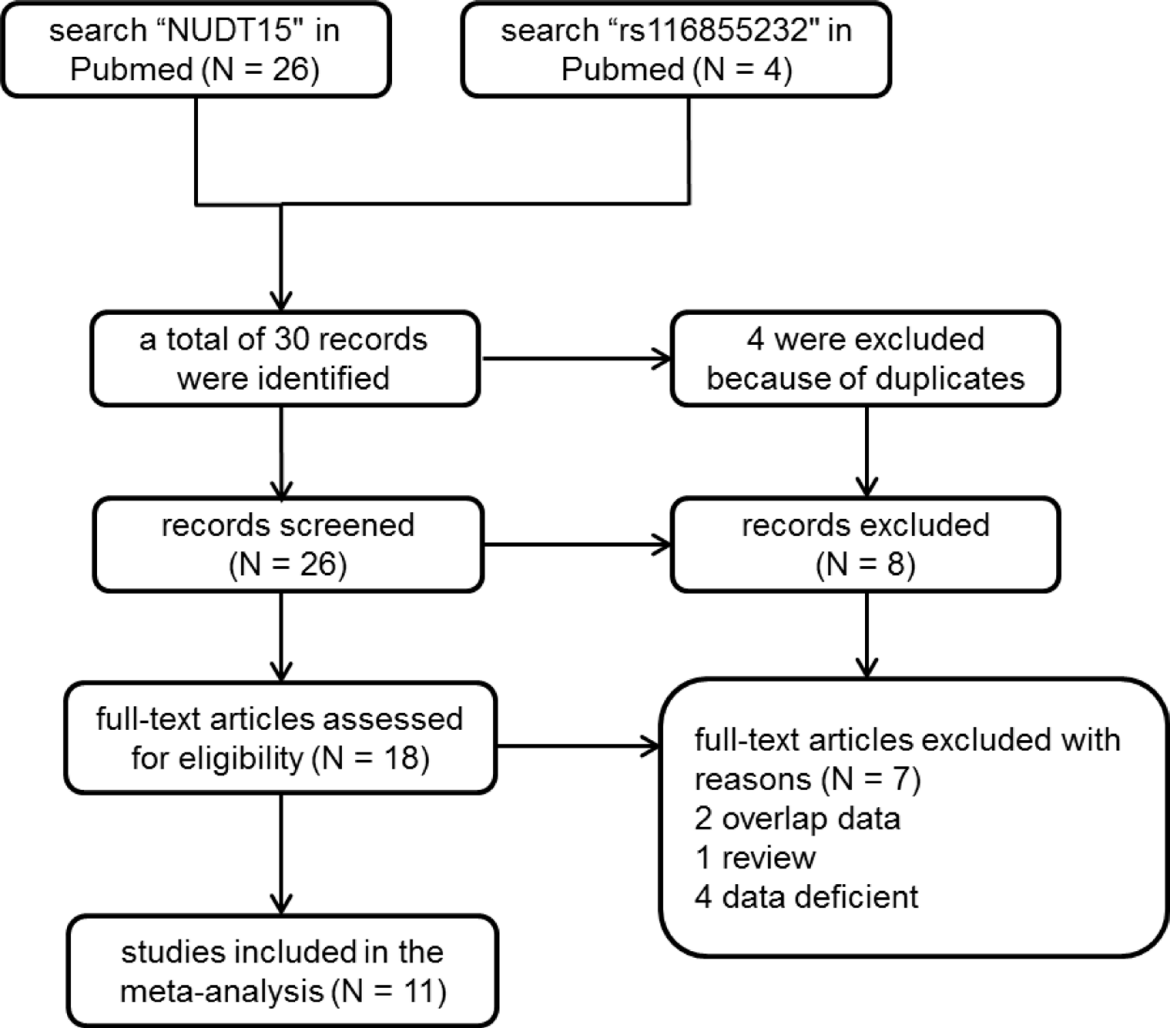
Flow chart of included studies for the meta-analysis

**Figure 2:**
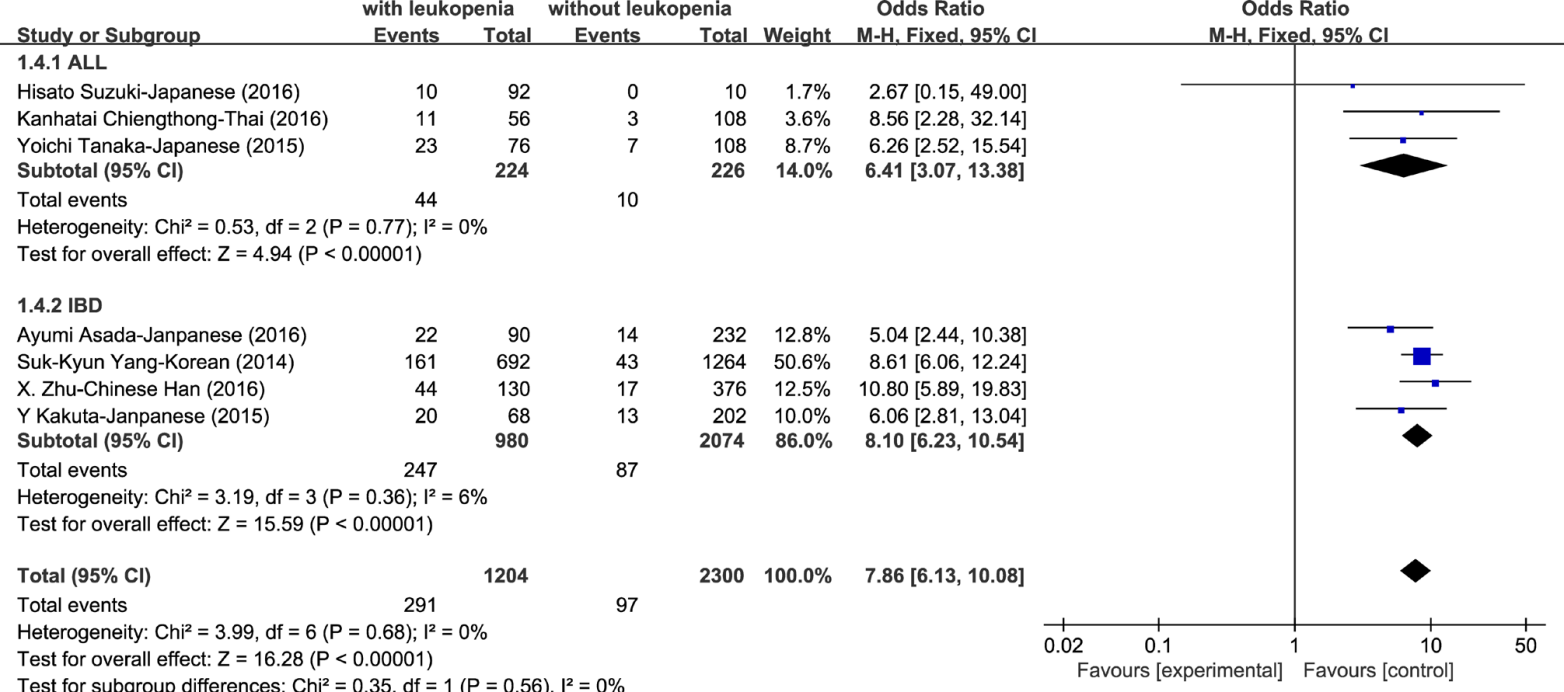
Forest plot of association of rs116855232 with thiopurines-induced leukopenia in allele model

**Figure 3:**
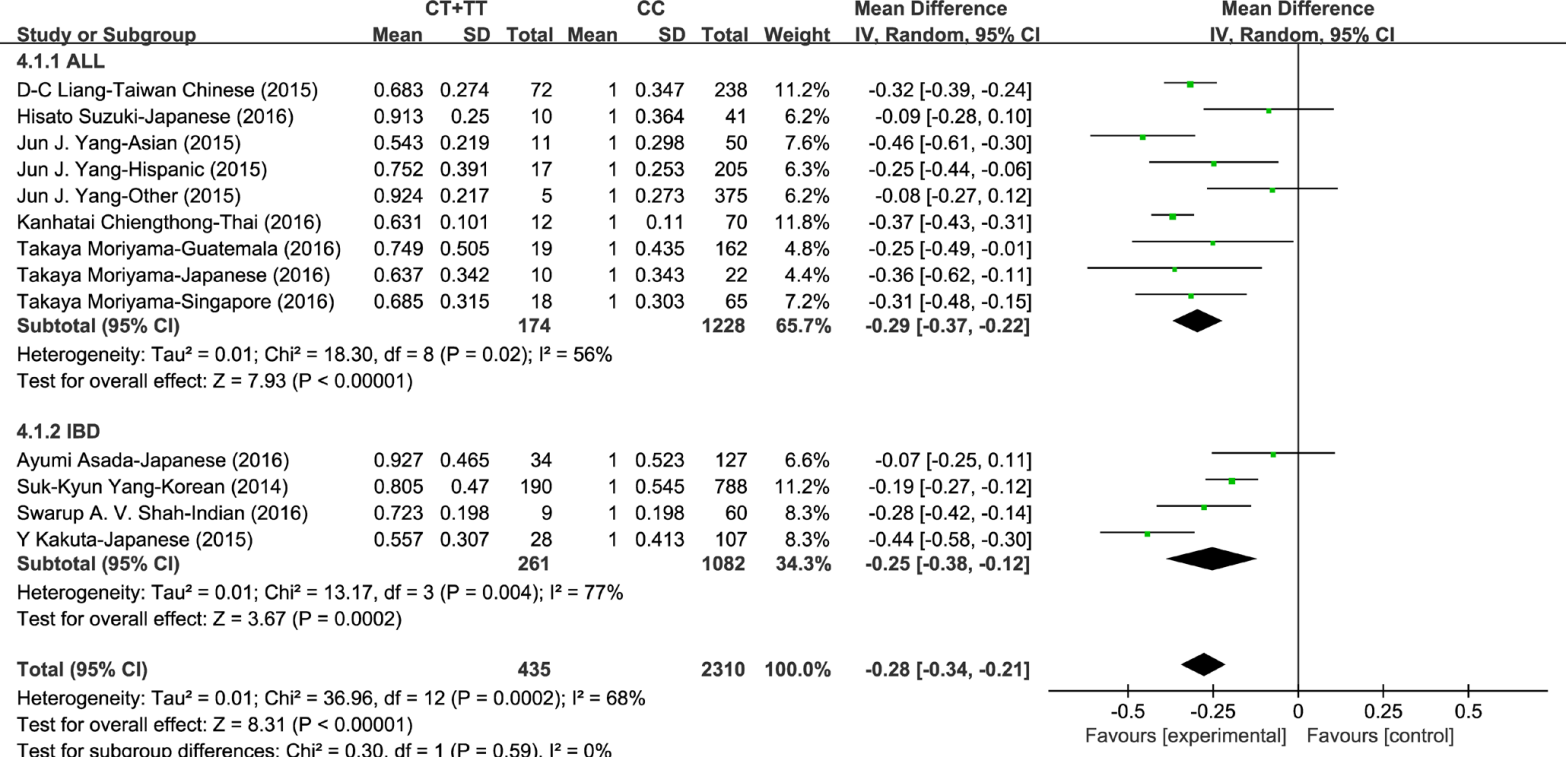
Forest plot of thiopurines intolerance dose associated with rs116855232 (T carriers compared to CC carriers)

Through literature searching (see Methods), 20 independent cohort studies that demonstrated in 11 articles met the inclusion criteria for meta-analysis (Figure 1). Characteristics of these studies were summarized in Table 1. We conducted meta-analyses on association of rs116855232 with thiopurines-induced myelotoxicity susceptibility, as well as thiopurines intolerance dose. First, 7 studies were included with a total of 602 cases (patients with thiopurines-induced myelotoxicity) and 1150 controls (patients without myelotoxicity) for myelotoxicity susceptibility analysis. Fixed effect model was used since no heterogeneity was observed in the allele model (*P* = 0.68, and *I*^2^ = 0%). Compared to C allele, variant T allele significantly exhibited a 7.86-fold (OR = 7.86, 95% CI: [6.13, 10.08]) increased risk to develop thiopurines-induced myelotoxicity in both IBD and ALL (*P* < 0.00001, Figure 2). Totally, the presence of rs116855232 variant allele had a sensitivity of 43.19% (260/602) and specificity of 91.74% (1055/1150) for all myelotoxicity events, while the specificity reached 84.59% (1323/1564) for early myelotoxicity events (Supplementary Tables 1 and 2). Additionally, Consistent association was also observed in dominant model (*P* < 0.00001, OR = 9.48, 95% CI: [7.20, 12.47]), and recessive model (*P* < 0.00001, OR = 18.10, 95% CI: [6.34, 51.68]). Secondly, 13 studies assessed the association between rs116855232 and thiopurines intolerance dose with a sample size of 2745. Random model was employed in dosage maintenance meta-analysis since the high heterogeneity among studies. Compared to CC carriers (as reference group), T allele carriers (CT and TT genotypes) required 28% (*P* < 0.00001, 95% CI: [–0.34, –0.21]) lower mean daily thiopurines dose. Because thiopurine dosage used in ALL patients is significantly higher than that in IBD patients, we separated the patients into two groups in terms of disease types, and found similar risk of thiopurines-induced myelotoxicity and thiopurine maintenance dosage reduction rate for T allele (Figure 3).

**Table 1:** Principle characteristics of the studies included in the Meta-Analysis for SNPs at NUDT15 rs116855232 locus

Year	Author [*]	Ethnicity	Sample sizea		Genotype counts (case)			Genotype counts (control)			AZA dose (mg/m2) (mean ± SD) (normalized dose)b			Diseases	Type of study
			Case	Control	TT	CT	CC	TT	CT	CC	TT	CT	CC		
2014	Suk-Kyun Yang [2]	Korean	346	632	14	133	199	0	43	589	2.335 ± 0.485 (0.522 ± 0.108)	3.697 ± 2.145 (0.827 ± 0.480)	4.472 ± 2.436 (1 ± 0.545)	IBD	myelotoxicity susceptibility and intolerance dose
2015	Y Kakuta [22]	Japanese	34	101	5	10	19	0	13	88	NA	1.613 ± 0.891 (0.557 ± 0.307)	2.915 ± 1.203 (1 ± 0.413)	IBD	myelotoxicity susceptibility and intolerance dose
2016	Ayumi Asada [21]	Japanese	45	116	2	18	25	0	14	102	2.12 (0.872)	2.26 ± 1.130 (0.930 ± 0.465)	2.43 ± 1.270 (1 ± 0.523)	IBD	myelotoxicity susceptibility and intolerance dose
2016	X. Zhu [27]	Chinese Han	65	188	4	36	25	0	17	171	NA	NA	NA	IBD	myelotoxicity susceptibility
2016	Swarup A. V. Shah [25]	Indian	6	63	1	5	0	0	3	60	2.066 ± 0.566 (0.723 ± 0.198)	2.858 ± 0.566 (1 ± 0.198)	IBD	intolerance dose	
2015	Jun J. Yang [12]	East Asian	61	NA	1	10	50	NA	NA	NA	10.125 (0.169)	35.55 ± 11.25 (0.594 ± 0.188)	59.85 ± 17.85 (1 ± 0.298)	ALL	intolerance dose
		Hispanic	222	NA	1	16	205	NA	NA	NA	2.175 (0.033)	52.425 ± 13.4 (0.796 ± 0.355)	65.85 ± 16.65 (1 ± 0.253 )		
		Other	380	NA	0	5	375	NA	NA	NA	NA	59.475 ± 13.95 (0.924 ± 0.217)	64.35 ± 17.55 (1 ± 0.273)		
2015	Yoichi Tanaka [14]	Japanese	38	54	5	13	20	1	5	48	NA	NA	NA	ALL	myelotoxicity susceptibility
2015	D-C Liang [23]	Taiwan Chinese	310	NA	2	70	238	NA	NA	NA	18.8 ± 7.4 (0.213 ± 0.084)	61.4 ± 23.4 (0.696 ± 0.265)	88.2 ± 30.6 (1 ± 0.347)	ALL	intolerance dose
2016	Kanhatai Chiengthong [28]	Thai	28	54	1	9	18	1	1	52	54.608 ± 8.719 (0.631 ± 0.101)	86.542 ± 9.525 (1 ± 0.110)	ALL	myelotoxicity susceptibility and intolerance dose	
2016	Takaya Moriyama [24]	Guatemala	181	NA	1	18	162	NA	NA	NA	8.944 (0.128)	54.954 ± 34.516 (0.789 ± 0.496)	69.638 ± 30.261 (1 ± 0.435)	ALL	intolerance dose
		Singaporean	83	NA	1	17	65	NA	NA	NA	5.522 (0.06)	65.894 ± 25.765 (0.721 ± 0.282)	91.354 ± 27.674 (1 ± 0.303)		
		Japanese	32	NA	1	9	22	NA	NA	NA	5.013 (0.05)	69.950 ± 28.912 (0.702 ± 0.290)	99.674 ± 34.231 (1 ± 0.343)		
2016	Hisato Suzuki [26]	Japanese	46	5	0	10	36	0	0	5	NA	59.946 ± 16.405 (0.913 ± 0.250)	65.647 ± 23.887 (1 ± 0.364)	ALL	myelotoxicity susceptibility and intolerance dose

^a^Case and Control indicates patients with or without thiopurines-induced myelotoxicity, respectively;

^b^6-MP dose was converted to AZA equivalent dose using a conversion factor of 2.08, Meeh-Rubner formula was used to unify the units into mg/m2, AZA dose of each genotype was also normalized against CC (wildtype) ;

*numbers in the brackets represent the references in the manuscript

NA: not available.

**Figure 4:**
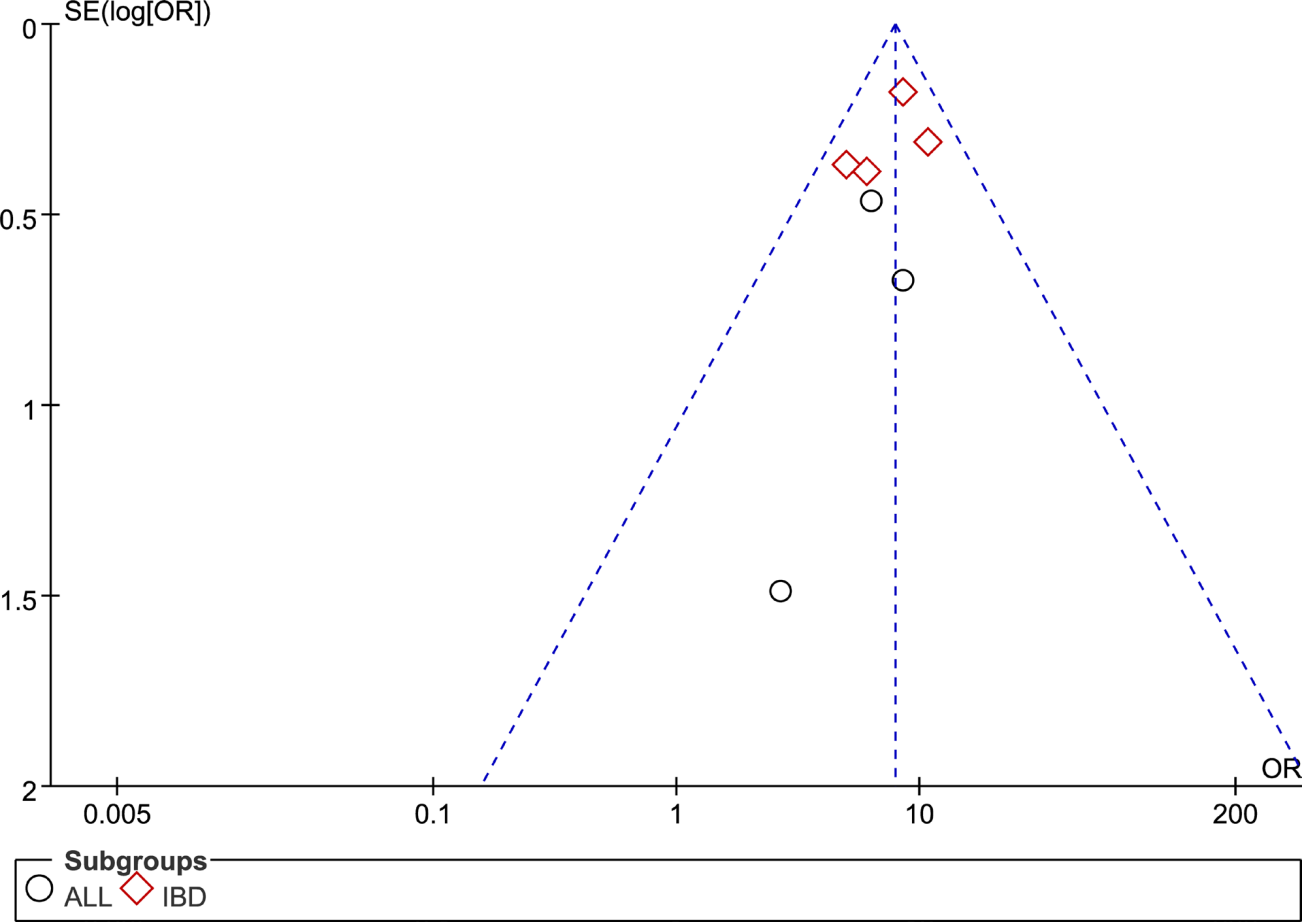
Funnel plot of publication bias test for association between rs116855232 polymorphism and thiopurines-induced myelotoxicity susceptibility

For both meta-analyses, we used Begg's test and Egger's test to measure the publication bias for all model, no evidence of obvious asymmetry was observed, such as Figure 4. Sensitivity analyses were also carried out by removing each study one at a time, the ORs remain stable, suggesting that the conclusion of rs116855232 impact on thiopurines-induced myelotoxicity susceptibility and thiopurines intolerance dose were robust and reliable.

### Crystal structure and protein stability prediction

**Figure 5:**
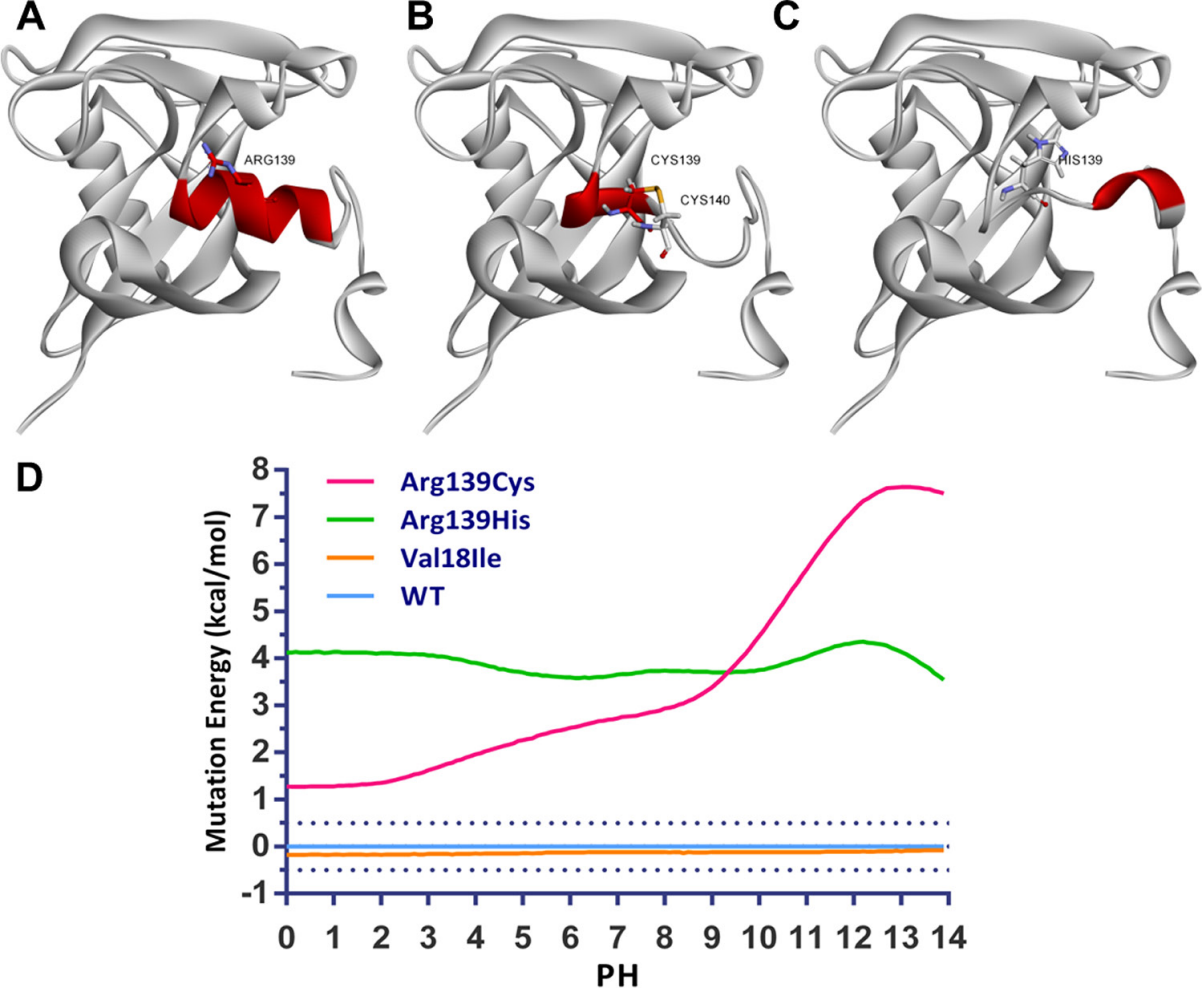
Impact of rs116855232 on protein structure and stability structures are illustrated for (**A**) wildtype, (**B**) Arg139Cys and (**C**) Arg139His for NUDT15, and subsequent protein stability has also been analyzed (**D**).

Besides rs116855232, two additional functional SNPs (i.e., rs186364861 [inducing Val18Ile], and rs147390019 [inducing Arg139His]) were also reported recently. To investigate the effect of the Val18Ile, Arg139His and Arg139Cys mutants on the protein function of NUDT15, we firstly constructed their mutant models through mutating selected residue of the crystal structure, and optimize the mutant region structure by using loop refinement. Through bioinformatics prediction, we noticed that Arg139His and Arg139Cys were located at the second α-helix, may perturb the α-helix loop and the base of the substrate binding pocket (Figure 5A, 5B, 5C). Additionally, Arg139Cys could lead to the formation of a disulfide bond with the adjacent cysteine residue (Cys140) that may further reduce the enzyme activity of NUDT15 (Figure 5B). However, Val18Ile mutant at the first β-sheet has less effect on the protein structure. Subsequently, mutant energy calculation showed similar results (data not shown). For protein stability estimation, mutant energy of Arg139His and Arg139Cys variants are greater than 0.5 kcal/mol, largely decrease the protein stability compared to wildtype and Val18Ile mutant, indicating the possible role of these genetic variants on NUDT15 function (Figure 5D).

### Cis-eQTL and epigenome regulation analyses for *NUDT15*

**Figure 6:**
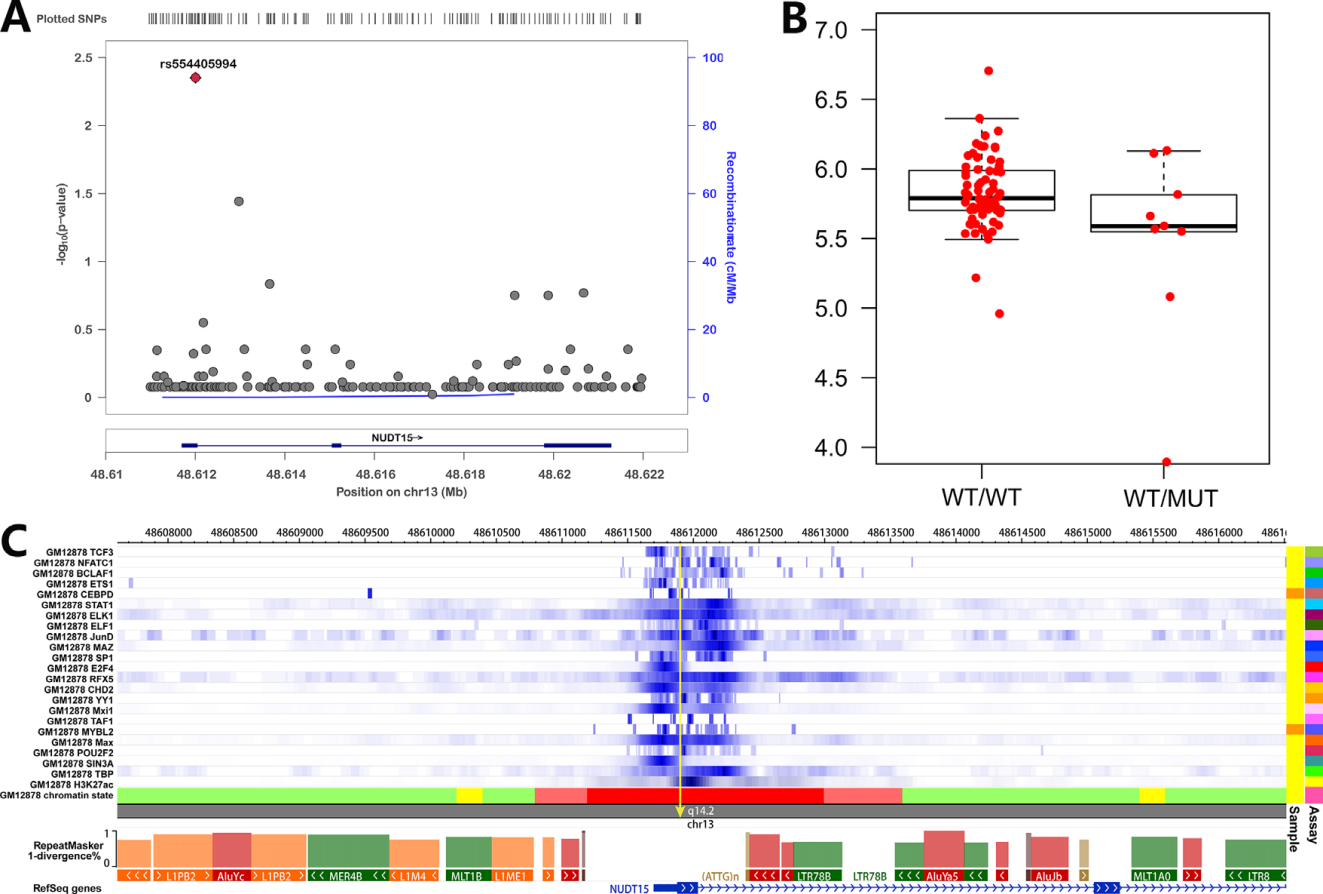
eQTL and epigenetic analysis for *NUDT15* SNPs (**A**) eQTL has been screened around NUDT15 locus and illustrated with online tool (i.e., LocusZoom); (**B**) association of NUDT15 expression with the top eQTL signal (rs554405994) was shown with boxplot; (**C**) Epigenomic signals at *NUDT15* locus was illustrated by using online tool (i.e., Epigenome Browser).

Although ~90% of early thiopurines-induced myelotoxicity can be predicted by rs116855232, sensitivity of such SNP for late myelotoxicity is still low, indicating more variants may be involved in such ADR. As down-regulation of *NUDT15* in cell lines can also sensitize leukemia cells to thiopurines [24], we thus conducted an eQTLs analyses for *NUDT15* to search additional potential pharmacogenetic markers in this gene. Expression level and SNP genotypes of LCLs were retrieved from the public resource (see Methods), and submitted for association analyses. Only one SNP (rs554405994) achieved statistical significance (*P* = 0.004) (Figure 6A), and LCLs from Asians with variant allele are related to lower *NUDT15* expression (Figure 6B). rs554405994 locates in the promoter region of *NUDT15*, with a high GC content and DNase I sensitivity. Multiple strong transcription factors and H3K27Ac binding signal can be detected around this rs554405994-located region according to the epigenomic information from public resource (i.e., Epigenome Browser [38], Figure 6C), indicating variant allele of rs554405994 may be associated with *NUDT15* expression through altering the binding affinity of transcription factors with gene promoter. Interestingly, rs554405994 also induces a GlyVal insertion between position 18 and 19 of NUDT15, and causes a slight reduction in NUDT15 activity according to the previous report [24]. Therefore, rs554405994 can impact thiopurine metabolism through both down-regulating gene expression and reducing enzyme activity, thus may be considered as a new causal variant for thiopurines-induced myelotoxicity.

## DISCUSSION

Ethnic diversity in genetics is an important factor for inherited predisposition to disease susceptibility, as well as drug treatment outcomes [39, 40]. For instance, SNPs at *ARID5B* locus are associated with ALL susceptibility with varied odds radio among ethnicities [41, 42], while missense SNP in *CDKN2A* exhibits significance only in Caucasians [43]. 6-MP is commonly used in ALL chemotherapy and can induce severe ADR events in some patients (mainly myelotoxicity), which can be largely explained by *TPMT* variants in Caucasians and blacks but not Asians due to the low variant allele frequency [15, 29]. Recently, missense SNP (i.e., rs116855232) in *NUDT15* has been linked to thiopurines-induced myelotoxicity in ALL as well as IBD by genome-wide association studies [2, 12]. Such association has been replicated in multiple independent follow-up studies by considering either myelotoxicity event or intolerance dose, and also exhibited ethnic specific mainly because the risk allele frequency is high in Asians and Hispanics (e.g., ~10% in Asians), but rare in Caucasians (0.2%) and not detected in blacks [12]. Therefore, rs116855232 of *NUDT15* testing is of greater diagnostic value than *TPMT* genotyping for prospective risk assessment of thiopurines-induced myelotoxicity in Asian population.

In this study, we conducted a meta-analysis to estimate the impact of rs116855232 in *NUDT15* on thiopurines-induced myelotoxicity as well as thiopurine intolerant. With allele model, T allele carriers have ~8-fold higher risk compared to C allele carriers to develop leucopenia no matter in ALL or IBD. However, patients with CT genotype have a significant lower myelotoxicity rate (229 out of 322, 71.10%) than TT genotype (31 out of 33, 93.94%), thus lower risk to develop leucopenia compared to CC genotype in additive model, with OR = 7.60 (95% CI: 5.77–10.03) and 18.10 (95% CI: 6.34–51.68), respectively. Moreover, the ADR rate varied largely in patients with CT genotype range from 43.48% to 100% (median = 72.22%), probably due to the starting dosage of thiopurines. Actually the incidence of thiopurines-induced myelotoxicity will increase with the higher standard dose (e.g., 35% vs. ~25% IBD patients suffered ADR in Suk-Kyun's study compared with others), but the T allele frequency are similar within patients with ADR among studies. However, statistical analysis can't be done due to the limited number of studies and complicated study design. For meta-analysis for thiopurine maintenance dose, high heterogeneity was observed in both IBD and ALL studies, possibly induced by drug response variations in patients with CT genotype as described above. However, we didn't use additive model to estimate the contribution of TT genotype to thiopurine intolerant compared to CC genotype, because 11 out of 13 studies have no more than one patient with TT genotype of rs116855232. Overall, our results indicate the important diagnostic value of rs116855232 in *NUDT15*, especially patients with TT genotype. Moreover, Suk-Kyun Yang et al demonstrated the significant higher diagnostic value of rs116855232 as indicator for early (OR = 35.13) than late leukopenia (OR = 5.29), with accordingly sensitivity of 89.40% (59/66) and 31.43% (88/280), respectively [2], suggesting not only the strong effect of rs116855232 on thiopurines-induced myelotoxicity, but also some other factors may be involved in late myelotoxicity events. Interestingly, additional SNPs in *NUDT15* described by Takaya et al are related to final maintenance thiopurines dosage, including rs147390019 (only common in Hispanic [1.7%]) and rs186364861 (only common in Asian [1.6%]) [24]. Compound heterozygote of these SNPs illustrated similar effect on thiopurines dosage adjustment as homozygous variant of rs116855232, indicating more attention should be paid for all functional *NUDT15* SNPs. However, meta-analysis is not applicable due to the lack of independent studies for these SNPs. In addition, interaction of *TPMT* and *NUDT15* variants have also been considered in some studies, heterozygous of functional SNPs of both genes show significant lower intolerant thiopurine dose than mono-heterozygous SNPs, but higher than homozygous variants in either SNPs [24]. However, more independent studies are needed for validation because the sample size of patients with either compound heterozygous of *NUDT15* variants or heterozygous genotype in both *TMPT* and *NUDT15* is small.

According to the previous report, NUDT15 is a nudix hydrolase which can degrade dGTP and dGDP *in vitro*, suggesting that it may reduce the active metabolites of thiopurine *in vivo* [44]. Indeed, loss-of-function of NUDT15 enzymatic activity induced by amino acid change of Arg139Cys (induced by rs116855232 risk allele), can largely explain thiopurines-induced myelotoxicity and thiopurines maintain dose. Other clinical relevant genetic variants were also conducted to estimate their impact on NUDT15 function. Interestingly, rs147390019 introduces amino acid change at the same position as rs116855232, resulting in Arg139His. As the crystal structure of wildtype NUDT15 and the importance of the position Arg139 in thiopurine has been characterized [45, 46], we further figured out that both Arg139Cys and Arg139His greatly impact on the crystal structure of NUDT15 by breaking α-helix of the active domain and reducing the stability of mutated NDUT15. However, it can't explain the effect of Val18Ile (induced by rs186364861) and Val18_Val19insGlyVal (induced by rs554405994) since little change has been seen in NUDT15 structure with these altered amino acids (data not shown). Due to the possibility of prediction error, experimental crystallization and structure analysis for mutated *NUDT15* is needed to determine the impact of these SNPs. Additionally, Takaya et al established stable cell lines and observed significant increase of TGTP/TGMP ratio as well as DNA-TG content in *NUDT15* knockdown cells [24], raising a possibility that eQTL for *NUDT15* may also be related to clinical ADR events. We thus conducted an eQTL analysis and screened out the top eQTL signal (i.e., rs554405994), which is predicted to be located at the promoter region with multiple binding sites of regulatory factors. Interestingly, this SNP has been described above as an inframe indel, inducing two amino acids (GlyVal) insertion between position 18 and 19. Therefore, the risk allele of rs554405994 is related to lower expression level and decreased enzyme activity of NUDT15 [24], thus could be considered as another pharmacogenetic marker for thiopurine response. However, further clinical confirmation studies are needed to determine the improved sensitivity for thiopurines-induced myelotoxicity after considering rs554405994, especially for the late ADR.

In conclusion, our meta-analysis demonstrates the strong significant association of the SNP (rs116855232) at *NUDT15* with thiopurines-induced myelotoxicity susceptibility and thiopurines intolerance dose in either ALL or IBD. Although rs116855232 has already been labeled as pharmacogenomics marker in PharmGKB (www.pharmgkb.org), we considered the recommended level could be upgrade from 1B to 1A for thiopurines usage. Besides, more independent studies are needed to estimate the impact other functional SNPs of *NUDT15* to guide the clinical usage of thiopurines.

## MATERIALS AND METHODS

### Literature and study acquisition

Systematically literature searching was carried out independently by two investigators from PubMed, Google Scholar and the Chinese National Knowledge Infrastructure (CNKI) date to July 27, 2016 according to following search terms: “rs116855232”, or “*NUDT15*”, or “thiopurine drugs” and “polymorphisms” and “*NUDT15*”, or “thiopurines” and “*NUDT15*”, or “polymorphism” and “*NUDT15*”. All papers were restricted to English (*N* = 30). Initially, checking of the titles as well as the abstracts was conducted to remove the duplicated articles along with papers that did not meet our subject. Then, we read through every remaining studies and retained valuable papers which meet the following criteria: (1) thiopurine drugs therapy based toxicity studies; (2) association of SNPs at *NUDT15* with thiopurine drugs intolerance or susceptibility to toxicity was evaluated; (3) information of patient number was provided, including patients with or without ADR, respectively; (4) provided the genotype counts or sufficient data to impute the genotypes, (5) data without overlap (*N* = 11). When multiple publications reported on the same or overlapping data, only the publication with the most updated or detailed data was included. The literature screening flow presented in Figure 1. Neither Ethical approval nor patient consent was needed, because all the information was acquired from published studies.

### Data extraction and verification

Using strict inclusion and exclusion criteria, detail information was extracted from each publications, including first author, ethnicity, sample size, and etc. 6-MP dose was converted to AZA equivalent dose using a conversion factor of 2.08 [2], and Meeh-Rubner formula was used to unify the units into mg/m^2^. Corresponding authors were contacted with if datasets were not accessible or incomplete for the required data. For accuracy, all the information was double checked and reviewed by another investigator. Detail information about the included papers was listed in Table 1 and Supplementary Tables 3, 4 and 5.

### Meta-analyses

By using Review Manager 5.3 software [34], we intended to analyze the association of rs116855232 polymorphism with thiopurine induced leukopenia susceptibility, or thiopurines intolerance dose, with allele model (variant T allele vs. wildtype C allele), dominant model (TT+TC vs.CC), and recessive model (TT vs. TC+CC). Carriers of rs116855232 CC was defined as a reference group in SNP genetic models, while CT or TT was defined as “rs116855232 T carriers”. To remove any heterogeneity caused by pharmacodynamic differences in thiopurine drugs sensitivity among the study populations, the thiopurine drugs maintenance dose (mean ± SD) in each genotype (including CT, TT, and T carriers, respectively) was normalized against the reference group [35]. Heterogeneity among those studies were evaluated by the Q statistic and the *I*^2^ statistic, of which Q approximately follows a *χ*^2^ distribution with k-1 (k indicates the number of studies) degrees of freedom. P value was used to detect the significance level of heterogeneity. *I*^2^ = (Q-(k-1))/Q*100%, ranging from 0–100%. *I*^2^ was considered as a critical value, when *I*^2^ < 50% and *P* > 0.1, fixed-effect model was used to calculate summary odds ratios (OR) and 95% confidence interval (95% CI), while the random-effect model should be employed under the circumstances of *I*^2^ > 50% and *P* < 0.1 because of high heterogeneity.

### Crystal structure prediction and protein stability estimation

The initial three dimensional geometric coordinates of the X-ray crystal structure of NUDT15 (PDB code: 5BON) were downloaded from the Protein Databank (PDB). The Val18Ile, Arg139His and Arg139Cys mutant models were constructed using the Build Mutants protocol of Discovery Studio 3.5 (Accelrys Inc., USA). Disulfide bridges of Arg139Cys mutant model was selected manually. Loop refinement protocol, which uses a looper algorithm to optimize the structure of a selected non-terminal loop region of a protein structure, was performed to evaluate the protein structure change of the mutants. Mutation energy (stability) was calculated using pH-dependent mode to investigate the effect of single-point mutations on protein stability.

### Cis-eQTL analyses

Expression level of *NUDT15* gene was obtained from public RNA-seq data resource of Lymphoblastoid cell lines for CHB/JPT (GSE11582) [36], and genotypes of SNPs (Chr13: 48582000-48622000, hg19 human genome version) around NUDT15 were obtained from the 1000 genome project website (http://browser.1000genomes.org/). SNPs with variant allele detected in less than three individuals were excluded. Genotype-expression association was assessed through a linear regression model for the available individuals (*N* = 447). Regional plots were constructed by plotting the negative logarithm of the P value for each SNP in a 11.2-kb window at the *NUDT15* locus using LocusZoom [37].

## SUPPLEMENTARY TABLES



## Abbreviations

AZA: azathioprine
6-MP: 6-mercaptopurine
IBD: inflammatory bowel diseases
ALL: acute lymphoblastic leukemia
SNP: single nucleotide polymorphism
95% CI: 95% confidence intervals
OR: odds ratios
eQTL: expression quantitative trait loci
CNKI: Chinese National Knowledge Infrastructure.
