# Supplementary material for: Impact of NUDT15 polymorphisms on thiopurines-induced myelotoxicity and thiopurines tolerance dose
Source: Oncotarget. 2017 Jan 11;8(8):13575–85. doi: 10.18632/oncotarget.14594 (PMC5355121; doi:10.18632/oncotarget.14594)
Supplement: Supplementary file 1 [file oncotarget-08-13575-s001.pdf]

Impact of *NUDT15* polymorphisms on thiopurines-induced myelotoxicity and thiopurines tolerance dose

Supplementary Materials

Supplementary Table 1: Calculation of sensitivity and specificity in all patients

Author [*]	TT Genotype Counts		CT Genotype Counts		CC Genotype Counts	
	No. Myelotoxicity[a]	No. No Myelotoxicity[b]	No. Myelotoxicity	No. No Myelotoxicity	No. Myelotoxicity	No. No Myelotoxicity
Yoichi Tanaka [14]	5	1	13	5	20	48
Kanhatai Chiengthong [28]	1	1	9	1	18	52
Hisato Suzuki [26]	0	0	10	0	36	5
Suk-Kyun Yang [2]	14	0	133	43	199	589
Y Kakuta [22]	5	0	10	13	19	88
Ayumi Asada [21]	2	0	18	14	25	102
X. Zhu [27]	4	0	36	17	25	171

[a]the number of patients with myelotoxicity.
[b]the number of patients without myelotoxicity.
[*]numbers in the brackets represent the references in the manuscript.

Supplementary Table 2: Calculation of sensitivity and specificity separately in early and late myelotoxicity patients

Author [*]	Myelotoxicity	TT Genotype Counts		CT Genotype Counts		CC Genotype Counts	
		No. Myelotoxicity[a]	No. No Myelotoxicity[b]	No. Myelotoxicity	No. No Myelotoxicity	No. Myelotoxicity	No. No Myelotoxicity
Yoichi Tanaka [14]	Early	5	1	5	13	12	56
	Late	0	1	8	5	8	48
Suk-Kyun Yang [2]	Early	14	0	45	131	7	781
	Late	0	0	88	43	192	589
Y Kakuta [22]	Early	5	0	4	19	1	106
	Late	0	0	6	12	18	75
Kanhatai Chiengthong [28]	Early	1	1	2	8	3	67
	Late	0	1	7	1	15	52
Ayumi Asada [21]	Early	2	0	2	30	2	125
	Late	0	0	16	14	23	102
X. Zhu [27]	Early	4	0	15	38	8	188
	Late	0	0	21	17	17	171

[a]the number of patients with myelotoxicity.
[b]the number of patients without myelotoxicity.
[*]numbers in the brackets represent the references in the manuscript.

Supplementary Table 3: Quality of the included studies

Article	Bias			
	Phenotype Definition	Genotyping	Population Stratification	Selective Reporting
Yoichi Tanaka (2015) [14]	Low	Low	Low	Low
Suk-Kyun Yang (2014) [2]	Low	Low	Low	Possible
D-C Liang (2015) [23]	Low	Low	Low	Possible
Jun J. Yang (2015) [12]	Low	Low	Low	Possible
Y Kakuta (2015) [22]	Low	Low	Low	Possible
Kanhatai Chiengthong (2016) [28]	Possible	Unknown	Low	Low
Ayumi Asada (2016) [21]	Low	Low	Low	Low
Takaya Moriyama (2016) [24]	Low	Low	Low	Low
X. Zhu (2016) [27]	Possible	Low	Low	Low
Swarup A. V. Shah (2016) [25]	Low	Low	Low	Low
Hisato Suzuki (2016) [26]	Low	Low	Possible	Low

Abbreviations: Low = low risk of bias; Possible = possible risk of bias; Unknown = no detail information.
*numbers in the brackets represent the references in the manuscript.

Supplementary Table 4: Hardy–Weinberg equilibrium of rs116855232 polymorphism and thiopurines-induced myelotoxicity susceptibility

Article [*]	Ethnicity	Case (Myelotoxicity)					Control (without Myelotoxicity)					HWE (P value)	
		TT	CT	CC	T allele	C allele	TT	CT	CC	T allele	C allele	Case	Control
Yoichi Tanaka (2015) [14]	Japanese	5	13	20	30	154	1	5	48	7	101	0.24	0.08
Suk-Kyun Yang (2014) [2]	Korean	14	133	199	161	531	0	43	589	43	1221	0.15	0.38
Y Kakuta (2015) [22]	Japanese	5	10	19	20	48	0	13	88	13	189	0.09	0.49
Kanhatai Chiengthong (2016) [28]	Thai	1	9	18	11	45	1	1	52	3	105	0.92	0.00
Ayumi Asada (2016) [21]	Japanese	2	18	25	22	68	0	14	102	14	218	0.58	0.49
X. Zhu (2016) [27]	Chinese Han	4	36	25	44	86	0	17	171	17	359	0.06	0.52
Hisato Suzuki (2016) [26]	Japanese	0	10	36	10	82	0	0	5	0	10	0.41	NA

Abbreviations: HWE = Hardy–Weinberg equilibrium.
*numbers in the brackets represent the references in the manuscript.
NA: not available.

Supplementary Table 5: Hardy–Weinberg equilibrium of rs116855232 polymorphism and thiopurines intolerance dose

Article [*]	Ethnicity	Case (Myelotoxicity)+Control (without Myelotoxicity)					HWE (*P* value)
		TT	CT	CC	T allele	C allele	
Suk-Kyun Yang (2014) [2]	Korean	14	176	788	204	1752	0.25
D-C Liang (2015) [23]	Taiwan Chinese	2	70	238	74	546	0.19
Jun J. Yang (2015) [12]	East Asian	1	10	50	12	110	0.55
	Hispanic	1	16	205	18	426	0.27
Y Kakuta (2015) [22]	Japanese	5	23	107	33	237	0.02
Kanhatai Chiengthong (2016) [28]	Thai	2	10	70	14	150	0.05
Ayumi Asada (2016) [21]	Japanese	2	32	127	36	286	0.99
Takaya Moriyama (2016) [24]	Guatemala	1	18	162	20	342	0.52
	Singaporean	1	17	65	19	147	0.92
	Japanese	1	9	22	11	53	0.95
Swarup A. V. Shah (2016) [25]	Indian	1	8	60	10	128	0.25
Hisato Suzuki (2016) [26]	Japanese	0	10	41	10	92	0.44

Abbreviations: HWE = Hardy–Weinberg equilibrium.
*numbers in the brackets represent the references in the manuscript.
